# Type 2 Diabetes Mellitus Exacerbates Brain Injury After *Status Epilepticus* in Rats

**DOI:** 10.3390/brainsci15111227

**Published:** 2025-11-15

**Authors:** Carol-Victoria Mérida-Portilla, Ángel Alberto Puig-Lagunes, Consuelo Morgado-Valle, Joel Martínez-Quiroz, Luis Beltrán-Parrazal, María-Leonor López-Meraz

**Affiliations:** 1 Doctorado en Investigaciones Cerebrales, Universidad Veracruzana, Xalapa 91190, Mexico; meridavictoria26@gmail.com; 2 Facultad de Medicina, Universidad Veracruzana, Minatitlán 96760, Mexico; anpuig@uv.mx; 3 Instituto de Investigaciones Cerebrales, Universidad Veracruzana, Xalapa 91190, Mexico; comorgado@uv.mx (C.M.-V.); lubeltran@uv.mx (L.B.-P.); 4 Facultad de Química Farmaceútica Biológica, Universidad Veracruzana, Xalapa 91000, Mexico; joemartinez@uv.mx

**Keywords:** streptozocin, diabetes mellitus, *status epilepticus*, neurodegeneration, injury

## Abstract

Background: Clinical and experimental evidence suggests comorbidity between diabetes mellitus (DM) and epilepsy, including a higher incidence of *status epilepticus* (SE). However, the association between Type 2 Diabetes Mellitus (T2DM) and epilepsy is not fully understood. Therefore, this study aimed to analyze the severity of SE and the consequent brain injury in male Wistar rats with T2DM. Methods: To induce T2DM, postnatal day (P) 3 rats were injected with streptozocin (STZ, 100 mg/kg, s.c.; n = 18); control rats received an equal volume of citrate buffer (pH 4.5) used as vehicle (n = 16). Glycemia was monitored at P30, P40, P60, and P90 in both experimental groups. Subsequently, rats were injected intraperitoneally with lithium chloride (LiCl, 3 mEq/kg, i.p.), and 18 h later, at P90, SE was induced by pilocarpine hydrochloride (30 mg/kg, s.c.). Matched control rats were injected with LiCl and physiological saline solution. The severity of SE, the neurodegeneration, cell and tissue loss, and microglia and glial responses were evaluated in the hippocampus, amygdala, thalamus, the piriform cortex. Results: Hyperglycemia was evident at P90 in STZ rats compared with vehicle (*p* < 0.05). T2DM rats had a higher frequency of stage V seizures and increased latency to the first stage V seizure and to SE compared with control rats (*p* < 0.05). T2DM rats showed an increased number of Fluoro-Jade B-positive cells, a reduction in cell density, and tissue loss, associated with an increased microglia density but a reduced glial cell count after SE (*p* < 0.05). Conclusions: Our findings suggest that T2DM is associated with greater seizure severity and increased brain injury following SE.

## 1. Introduction

Growing evidence suggests a bidirectional relationship between diabetes mellitus (DM) and epilepsy [1,2,3]. DM is a metabolic disease characterized primarily by a chronic increase in blood glucose levels due to insufficient insulin secretion, impaired insulin action, or both [4]. Epilepsy is a brain disorder characterized by a chronic predisposition to seizures, resulting from excessive and hypersynchronous abnormal neuronal activity [5,6]. Seizures can result from a metabolic disorder [7,8], and it has been estimated that up to 25% of diabetic patients may develop a seizure during the course of the disease [1]. T2DM has been reported to increase the risk of developing epilepsy by approximately 1.6-fold compared with healthy individuals [2].

Some of the first reports of this association describe partial seizures in children with hyperglycemia without ketoacidosis [9] and children with well-controlled epilepsy who had partial seizures resistant to antiseizure medication and were subsequently diagnosed with insulin-dependent type 1 DM (T1DM) [10].

Reports also describe middle-aged to older adults presenting with focal seizures, generalized tonic–clonic seizures, *epilepsia partialis* continua, or *status epilepticus* (SE) associated with uncontrolled chronic hyperglycemia, both in people with non-insulin-dependent type 2 DM (T2DM), and in those without previously diagnosed DM. A history of poor chronic glycemic control, reflected by elevated HbA1c levels, is associated with increased seizure severity, leading to significantly higher rates of seizure clustering and secondary generalization compared to non-diabetic people [11]. Notably, this study estimated that approximately 19–25% of patients with T2DM have experienced seizures, with about 15% having at least one episode of SE [11]. Furthermore, glycemic derangement appears to play a critical role in seizure control, as high plasma glucose levels have been identified as an independent risk factor for uncontrolled seizures in people experiencing SE. In a study of 211 patients with SE, hyperglycemia was considered a significant contributing factor to refractory SE, with each milligram increase in plasma glucose associated with a 1% higher possibility of uncontrolled SE [12].

Clinical manifestations of this metabolic disorder include focal seizures and *epilepsia partialis continua*, which often emerges as the initial presentation of previously undiagnosed diabetes [13,14,15]. These non-ketotic hyperglycemia-related seizures may be refractory to traditional antiseizure therapy, yet they tend to resolve rapidly following correction of hyperglycemia with rehydration and insulin therapy [13,14,15]. While some cases represent a purely transient and reversible neuroendocrine condition, sometimes accompanied by transient focal brain lesions on MRI [15], other studies suggest that non-ketotic hyperglycemia-associated seizures may also unmask underlying acute structural pathologies, such as ischemic or hemorrhagic stroke, or pre-existing vascular epilepsy [16,17].

Preclinical research has also shown that T1DM induced in adult rats or mice by the administration of streptozocin (STZ), a toxin that primarily affects the β-cells of the pancreas [18,19], increases seizure severity, produces spatial memory deficits, and causes neuronal loss in the hippocampus [20,21]. Similarly, T2DM induced by neonatal STZ promotes higher seizure severity and neurodegeneration after SE in the hippocampus, amygdala, thalamus and pyriform cortex of adult rats [22]. However, to date, the association between T2DM and epilepsy remains incompletely understood. Therefore, this study aimed to analyze brain injury, glia and microglia responses following lithium-pilocarpine SE in adult rats with T2DM induced by neonatal application of STZ.

## 2. Materials and Methods

### 2.1. Animals

All experiments were conducted following the Mexican Official Norm on Care and Use of Laboratory Animals (NOM-ZOO-062-1999). The experimental protocol was approved by the Animal Care Committee of the Instituto de Investigaciones Cerebrales, Universidad Veracruzana (2019-003-b). Male Wistar rats derived from our breeding colony were housed in our facilities at 22–25 °C temperature, 65–75% relative humidity, and a 12 h light-dark (lights on at 08:00 h). The day of birth was designated the postnatal day (P) zero. Litters were adjusted to eight pups, and the rats were kept with their mothers until weaning on postnatal day 21. Thereafter, rats were housed in groups of four to five until adulthood, with free access to water and food (Lab Diet, St. Louis, MO, USA).

### 2.2. Type 2 Diabetes Mellitus Model

On postnatal day 3 (P3), rats were subcutaneously injected either with STZ (100 mg/kg, diluted in 0.1M citrate buffer pH 4.5, used as vehicle) (n = 18) or vehicle alone (n = 16) [22]. Blood glucose levels were measured prior to STZ or vehicle injection and subsequently at postnatal days 30 (P30), 40 (P40), 60 (P60), and 90 (P90). For this purpose, a blood drop was obtained from the tail vein (after asepsis of the sampling area), and glucose concentration was determined using a OneTouch UltraMini model glucometer (LifeScan, Milpitas, CA, USA). Blood samples were collected after a 5 h fasting period.

### 2.3. Status Epilepticus Model

On the postnatal day 89 (P89), rats received intraperitoneal injections of lithium chloride (LiCl; 127.2 mg/Kg. diluted in sterile water; Sigma, St. Louis, MO, USA). Eighteen h later, on P90, SE was induced by subcutaneous injection of pilocarpine hydrochloride (30 mg/kg; Sigma, St. Louis, MO, USA) diluted in physiological saline (STZ + SE, n = 10). The control group (Veh + SE, n = 12) received LiCl followed by physiological saline instead of pilocarpine, to ensure equivalent handling. Behavioral motor seizures were carefully monitored and scored according to the Racine scale [23]: 1 = Mouth and facial movements; 2 = Head nodding; 3 = Forelimb clonus; 4 = Rearing; 5 = Rearing and falling. Only animals reaching SE, defined as near-continuous seizure activity lasting 30 min [24], were included in the study. One hour after SE onset, rats received diazepam (10 mg/Kg, i.p) to improve survival and isotonic saline (s.c.) to avoid dehydration. All lithium-pilocarpine-treated rats had SE.

### 2.4. Experimental Groups for Histological Analysis

To analyze brain injury, glia and microglia responses following lithium-pilocarpine SE rats were randomly assigned to four experimental groups: (1) Veh, rats injected with citrate buffer on P3, with LiCl on P89, and with saline on P90 (n = 8); (2) STZ, rats injected with STZ on P3, with LiCl on P89, and saline on P90 (n = 8); (3) Veh + SE, rats injected with citrate buffer on P3, with LiCl on P89 and pilocarpine on P90 to induce SE (n = 8); (4) STZ + SE, rats injected with STZ on P3, and with LiCl on P89 and pilocarpine on P90 to induce SE (n = 10).

### 2.5. Tissue Processing for Histological Analysis

All rats were anesthetized with an overdose of pentobarbital (120 mg/kg, i.p.) 24 h after SE or control treatment. Subsequently, rats underwent transcardial perfusion with saline (0.9% NaCl; Sigma) followed by 4% phosphate-buffered paraformaldehyde (Sigma, St. Louis, MO, USA). Brains were kept in situ at 4 °C overnight and then removed and postfixed in the same fixative for 2 h. Brains were dehydrated, embedded in paraffin (Paraplast; Sigma, St. Louis, MO, USA), and cut into 10-μm-thick coronal sections at the level of the dorsal hippocampus. Sections were deparaffinized, rehydrated through graded ethanol series, and finally placed in 0.1 M phosphate buffer (PB) before any procedures.

### 2.6. Fluoro-Jade B Staining

Neurodegeneration after SE was evaluated using Fluoro-Jade B (F-JB, Chemicon, Temecula, CA, USA, AG310) staining [25]. Sections were incubated with 0.06% KMnO_4_ (Sigma) followed by 0.001% F-JB (30 min in each solution) and examined using an Olympus AX70 fluorescence microscope (Nagano, Japan) equipped with selective excitation and emission filters.

### 2.7. Hematoxylin and Eosin Staining (H&E)

Brain sections were stained with H&E staining using Harris’ hematoxylin (Sigma) and 0.01% acidic alcoholic eosin solution (Sigma, St. Louis, MO, USA) [26]. Sections were dehydrated in ethanol and xylene and mounted with a non-aqueous medium (Permount; Fisher Scientific, Fair Lawn, NJ, USA).

### 2.8. Immunohistochemical Detection of Glia and Microglia

Sections underwent antigen retrieval in 10 mM citrate solution (pH 6.0), were washed in 0.1 M PB, and endogenous peroxidases were quenched with H_2_O_2_ and methanol. After washing in 0.1 M PB and 0.1 M PB containing 1% triton X-100 (1% PBT), sections were incubated in blocking solution (1% horse serum in 0.1 M PB or 5% rabbit serum in 0.1 M PB) for 1 h. Primary antibodies were applied overnight at 4 °C: anti-Glial Fibrillary Acidic Protein (GFAP, 1:500; Sigma-Aldrich, St. Louis, MO, USA; ZRB2383) and anti-ionized calcium-binding adapter molecule 1 (Iba-1, 1:500; Abcam, Cambridge, UK; ab107159). After washing in 0.1 M PB, sections were incubated with biotinylated secondary antibodies (1:400, Vector BA1000 anti-rabbit antibody or 1:200, Sigma-Aldrich A5420 anti-goat antibody) for 2 h, followed by the ABC-peroxidase complex (VECTASTAIN Elite ABC HRP kit, Newark, CA, USA) for 90 min. Staining was revealed with diaminobenzidine (3,3′-Diaminobenzidine Peroxidase Substrate Kit; Vector, Newark, CA, USA) and sections were mounted with a non-aqueous mounting medium (Fluoromount, Sigma, St. Louis, MO, USA).

### 2.9. Cell Counting and Tissue Loss Analysis

Histological analyses were performed bilaterally in the CA1 and CA3 pyramidal layers of the dorsal hippocampus, the piriform cortex (Pir, layer II), the basolateral amygdala nucleus (BLA), and the dorsomedial thalamic nucleus (DMT). Regions were consistently defined according to the stereotaxic coordinates provided in the rat brain atlas [27] (3–3.24 mm posterior to bregma). These areas were selected because they exhibit extensive neuronal damage after SE [28,29]. Digital images were acquired using a Leica DM500 microscope equipped with a digital camera ICC50 HD (Leica microsystem, Wetzlar, Germany) or an Olympus AX 70 epifluorescence microscope (Nagano, Japan) equipped with a U-MWB cube (450–480 nm, Olympus, Nagano, Japan) and a digital camera Leica DFC450 C (Leica microsystem, Wetzlar, Germany).

For H&E-stained sections, cell nuclei with clear morphology were identified within defined region of interest (ROI). Astrocytes and microglia (GFAP and Iba-1 immunoreactive cells, respectively) were identified based on morphology and DAB staining intensity thresholds using Fiji software (ImageJ v2.1.0/1.53c) [30]. ROI sizes were 20,000 μm^2^ (10,000 μm^2^ per hemisphere) for CA1, CA3 and Pir, and 80,000 μm^2^ (40,000 μm^2^ per hemisphere) for BLA and DMT. F-JB–positive cells, characterized by green fluorescence, were identified according to fluorescence intensity thresholds, using Fiji. ROI sizes were 12,000 μm^2^ (6000 μm^2^ per hemisphere) for CA1, CA3 and Pir, and 70,000 μm^2^ (35,000 μm^2^ per hemisphere) for BLA and DMT. Semi-automatic cell counting was performed with Fiji, with manual verification to avoid false positives. The final data per animal was obtained by averaging counts from 4 consecutive sections.

Tissue loss, referred to as tissue disintegration by Kalani et al. [31], was defined as areas within the ROI lacking cellular or neuropil staining, confirmed across consecutive sections, indirectly suggesting edema [28,29,31,32]. Vacuolization or parallel spaces likely attributable to processing artifacts were excluded from the analysis. This assessment was performed in Fiji on the same photomicrographs and ROIs used for H&E-based cell counting. Tissue loss was expressed as a percentage of the total ROI area, and the final value per animal was obtained by averaging measurements from four consecutive sections. Some brain sections, even under control conditions (Veh and STZ groups), displayed occasional processing-related spaces rather than pathological tissue loss, which may represent a limitation of our study.

All histological evaluations were conducted by experimenters blinded to the treatment groups.

### 2.10. Statistical Analysis

Data normality was assessed using the Shapiro–Wilk test. When the data did not follow a normal distribution (*p* < 0.05), nonparametric tests were used for subsequent analyses. Blood glucose levels were analyzed with a two-way repeated-measures Analysis of Variance (ANOVA) with two factors: (1) Treatment (Two levels: vehicle and STZ) and (2) Time of evaluation (four levels: 30, 40, 60, and 90 postnatal days); post hoc pairwise comparisons using the Bonferroni correction were conducted only when the interaction term reached statistical significance. Reported *p*-values were adjusted for multiple comparisons. Seizure behavioral parameters and the number of F-JB positive cells were evaluated with a nonparametric Mann–Whitney U test for independent samples. Histological parameters, including the number of cells, percentage of tissue loss, and numbers of glial and microglial cells, were analyzed with a two-way ANOVA with two factors: (1) Treatment (Two levels: Vehicle and STZ) and (2) SE induction (Two levels: No SE and SE) followed by a Bonferroni post hoc test with correction for multiple comparisons using statistical hypothesis testing. The significance level was set at *p* < 0.05. Analyses and graphs were generated using StatPlus v8 (www.analystsoft.com/es/; accessed on 28 June 2021) or Prisma GraphPad v10.5. Data analyzed by ANOVA are expressed as mean ± SEM, while data analyzed nonparametrically are expressed as median (interquartile range, IQR).

## 3. Results

### 3.1. Blood Glucose Levels

The two-way ANOVA revealed significant effects of treatment (F(1,32) = 70.84, *p* < 0.0001), time of evaluation (F(3,96) = 14.67, *p* < 0.0001), and their interaction (F(3,96) = 17.63, *p* < 0.0001) on blood glucose levels. Post hoc analysis showed a significant increase in glycemia in STZ-treated rats at P90 (296.2 ± 26.2 mg/dL) compared with the Vehicle-treated rats (110 ± 3.4 mg/dL) (*p* < 0.0001). STZ-treated rats also exhibited higher glycemia at P90 relative to P30 (155.7 ± 4 mg/dL), P40 (166.9 ± 6.5 mg/dL) and P60 (188.6 ± 16.8 mg/dL) (*p* < 0.0001). Vehicle-treated rats remained normoglycemic throughout the study. These results support the development of diabetic hyperglycemia in neonatally STZ-injected rats, consistent with previous reports [22].

### 3.2. Status Epilepticus Severity

The STZ + SE group showed a significant increase in total number of generalized seizures (stage IV and V) (median [IQR] = 6.5 [2.25]) compared with the Veh + SE group (median [IQR] = 3 [3.0]) (MWU = 14, *p* < 0.005). This increase was due to a higher number of stage V seizures in the STZ + SE group (median [IQR]= 5 [1.5]) then in the Veh + SE group (median [IQR] = 2 [2.0]) (MWU = 2, *p* < 0.002), whereas the number of phase IV seizures was similar between groups (median [IQR] = 0.5 [1.25] in STZ + SE group and 2 [1] in Veh + SE group) (MWU = 31.5, *p* = 0.051). The average duration of stage V seizures did not differ significantly between groups (STZ + SE: median [IQR] = 0.9 [0.5] min; Veh + SE: 0.8 [0.62] min) (MWU = 37, *p* = 0.13). However, latency to the first generalized seizure (stage IV or V) was longer in the STZ + SE group (median [IQR] = 42.4 [4.36] min) compared with the Veh + SE group (median [IQR] = 21.3 [2.76] min) (MWU = 0, *p* < 0.002). Similarly, latency to SE onset was increased in the STZ + SE group (median [IQR] = 47.7 [8.03] min) with respect to the Veh + SE group (median [IQR] = 26.8 [4.77] min) (MWU = 0, *p* < 0.002). Recovery time after diazepam administration to stop SE was approximately 1 h and did not differ between groups (STZ + SE: median [IQR] = 63.9 [10.62] min; Veh + SE: median [IQR] = 58.85 [13.32] min) (MWU = 38.5, *p* = 0.16). The results suggest that rats with T2DM experience SE of greater severity.

### 3.3. Neurodegeneration

**Figure 1:**
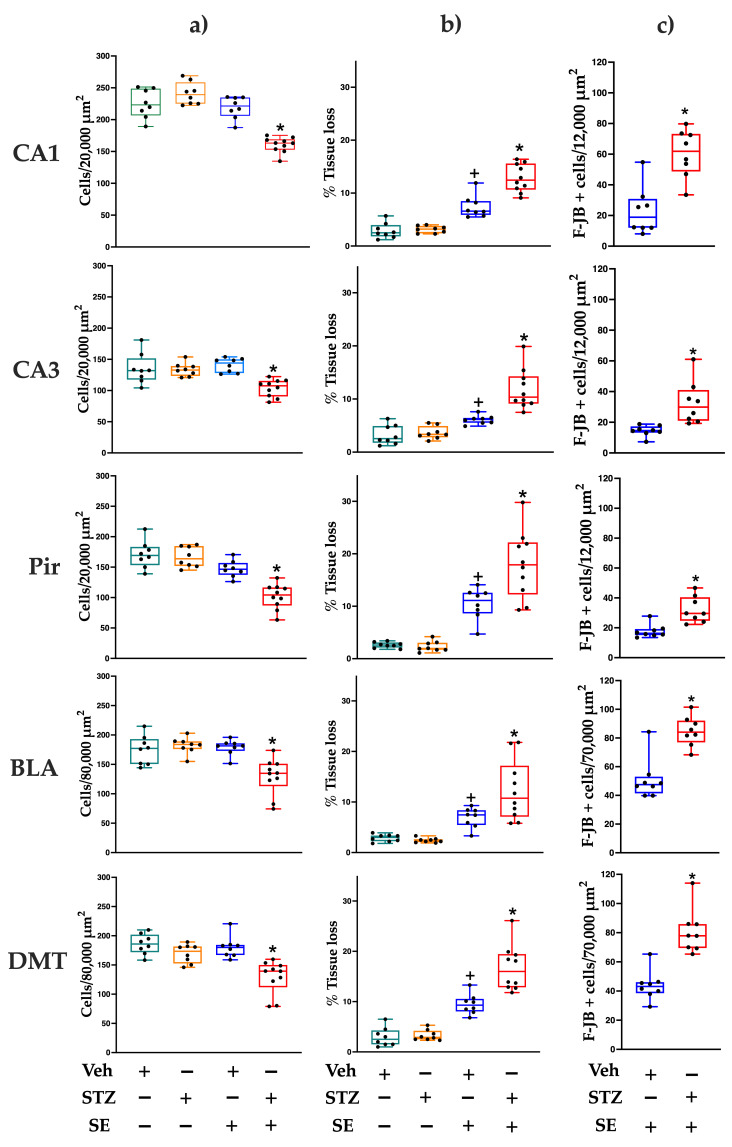
Number of H&E-stained cells (**a**), percentage of tissue loss (**b**), and number of F-JB positive cells (**c**) in CA1 and CA3 regions of the dorsal hippocampus, piriform cortex (Pir), basolateral amygdala (BLA), and dorsomedial thalamus (DMT) of rats treated with vehicle (Veh) or streptozotocin (STZ), with or without *status epilepticus* (SE). Data represent medians and interquartile ranges; whiskers indicate minimum and maximum values for each group. Individual data points represent each rat (n = 8 Veh, 8 STZ, 8 Veh + SE and 10 STZ + SE). * *p* < 0.05 vs. Veh, STZ, and Veh + SE; + *p* < 0.05 vs. Veh and STZ.

Rats from the Veh and STZ groups did not show F-JB positive cells in any brain region; therefore, neurodegeneration was only evaluated in those rats that developed SE (Veh + SE and STZ + SE). The STZ + SE group showed a higher number of F-JB positive cells in the hippocampal CA1 (median [IQR] = 61.9 [24.6]; MWU = 4, *p* < 0.005) and CA3 (median [IQR] = 29.2 [20.1]; MWU = 0, *p* < 0.005) fields, Pir (median [IQR] = 29.6 [15.8]; MWU = 3, *p* < 0.005), BLA (median [IQR] = 84.1 [15.2]; MWU = 4, *p* < 0.005) and DMT (median [IQR] = 77.9 [16.5]; UMW = 0, *p* < 0.005) compared with the Veh + SE group (median [IQR] = 18.9 [18.9], 14.6 [4.2], 16.3 [4.07], 47.5 [11.7], 43.2 [7.6], respectively) (Figure 1). The results show that SE induces more extensive neurodegeneration in rats with T2DM.

### 3.4. Cell Counting and Tissue Loss

**Figure 2:**
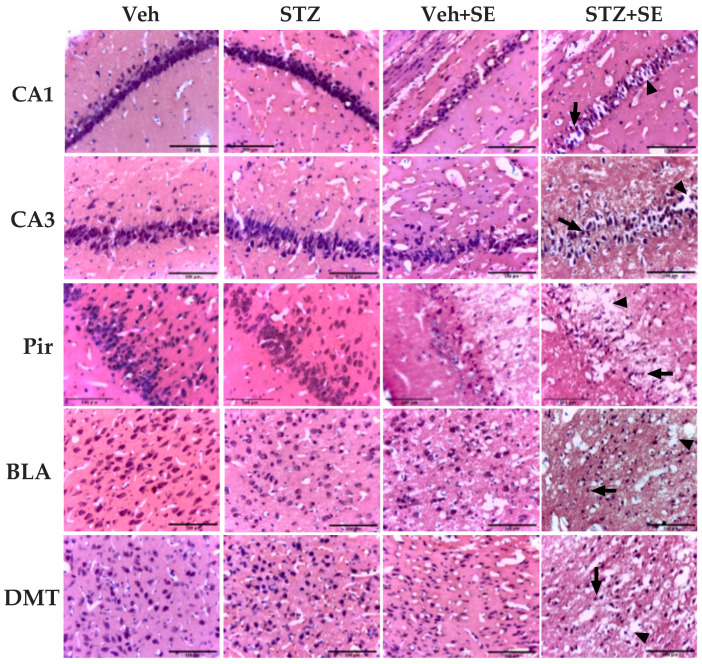
Representative photomicrographs (40×) of CA1 and CA3 regions of the hippocampus, piriform cortex (Pir), basolateral nucleus of the amygdala (BLA), and dorsomedial nucleus of the thalamus (DMT), stained with hematoxylin and eosin, from rats treated with vehicle (Veh) or streptozotocin (STZ), with or without *status epilepticus* (SE). STZ + SE rats show reduced cell numbers in all regions compared with Veh, STZ, and Veh + SE groups. Cells in SE groups exhibit shrunken somata and pyknotic nuclei (arrows). Tissue loss, indicated by areas lacking cellular or neuropil staining (arrowheads), is evident in rats that experienced SE and is most pronounced in STZ + SE rats. Scale bar = 100 µm.

The aim of our study was to determine whether T2DM exacerbates the severity and brain injury induced by SE. The two-way ANOVA revealed statistically significant main effects of STZ treatment (CA1: F(1,30) = 18.44, *p* < 0.001, ηp^2^ = 0.294; CA3: F(1,30) = 15.6, *p* < 0.05, ηp^2^ = 0.289; Pir: F(1,30) = 18.7, *p* < 0.001, ηp^2^ = 0.328; BLA: F(1,30) = 9.58, *p* < 0.005, ηp^2^ = 0.197; DMT: F(1,30) = 23.95, *p* < 0.001, ηp^2^ = 0.414) and SE (CA1: F(1,30) = 60.48, *p* < 0.0001, ηp^2^ = 0.634; CA3: F(1,30) = 5.65, *p* < 0.05, ηp^2^ = 0.136; Pir: F(1,30) = 57.60, *p* < 0.0001, ηp^2^ = 0.607; BLA: F(1,30) = 12.12, *p* < 0.005 ηp^2^ = 0.244; DMT: F(1,30) = 12.17, *p* < 0.0001, ηp^2^ = 0.241) on cell counts across all regions. Importantly, there was a statistically significant interaction between STZ treatment and SE (CA1: F(1,30) = 35.12, *p* < 0.0001, ηp^2^ = 0.563; CA3: F(1,30) = 8.39, *p* < 0.05, ηp^2^ = 0.260; Pir: F(1,30) = 5.98, *p* < 0.05, ηp^2^ = 0.253; BLA: F(1,30) = 12.41, *p* < 0.005, ηp^2^ = 0.311; DMT: F(1,30) = 3.64, *p* < 0.05, ηp^2^ = 0.156). Post hoc comparisons confirmed significantly fewer cells in the CA1, CA3, Pir, BLA, and DMT regions of the STZ + SE group compared with the Veh, STZ, and Veh + SE groups (*p* < 0.002 for all regions), as illustrated in Figure 1 and Figure 2, indicating that the combined condition of T2DM and SE produced a greater reduction in cell number than would be expected from their independent effects.

Analysis of tissue integrity revealed statistically significant main effects of STZ treatment (CA1: F(1,30) = 25.35, *p* < 0.0001, ηp^2^ = 0.378; CA3: F(1,30) = 50.33, *p* < 0.0001, ηp^2^ = 0.322; Pir: F(1,30) = 11.50, *p* < 0.05, ηp^2^ = 0.199; BLA: F(1,30) = 6.42, *p* < 0.05, ηp^2^ = 0.124; DMT: F(1,30) = 21.61, *p* < 0.05, ηp^2^ = 0.331) and SE (CA1: F(1,30) = 123.98, *p* < 0.0001, ηp^2^ = 0.795; CA3: F(1,30) = 21.80, *p* < 0.0001, η^2^ = 0.606; Pir: F(1,30) = 85.39, *p* < 0.0001, ηp^2^ = 0.726; BLA: F(1,30) = 38.27, *p* < 0.0001, ηp^2^ = 0.537; DMT: F(1,30) = 112.78, *p* < 0.0001, ηp^2^ = 0.776) on tissue loss across all regions analyzed, with significant interaction between STZ treatment and SE: CA1 (F(1,30) = 9.62, *p* < 0.005, ηp^2^ = 0.340), CA3 (F(1,30) = 5.12, *p* < 0.05, ηp^2^ = 0.266), Pir (F(1,30) = 5.51, *p* < 0.05, ηp^2^ = 0.224), BLA (F(1,30) = 4.47, *p* < 0.05, ηp^2^ = 0.167), and DMT (F(1,30) = 7.16, *p* < 0.05, ηp^2^ = 0.290) regions. Multiple comparisons confirmed a significantly greater percentage of tissue loss in the STZ + SE group compared with all other groups (*p* < 0.0005 for all regions). These findings demonstrate that both T2DM and SE independently contribute to brain tissue damage, but their combined occurrence produces a markedly greater injury. Moreover, tissue loss was significantly higher in the Veh + SE group than in the Veh and STZ groups (*p* < 0.005 for all regions), as shown in Figure 1 and Figure 2.

### 3.5. Glia and Microglia Counting

**Figure 3:**
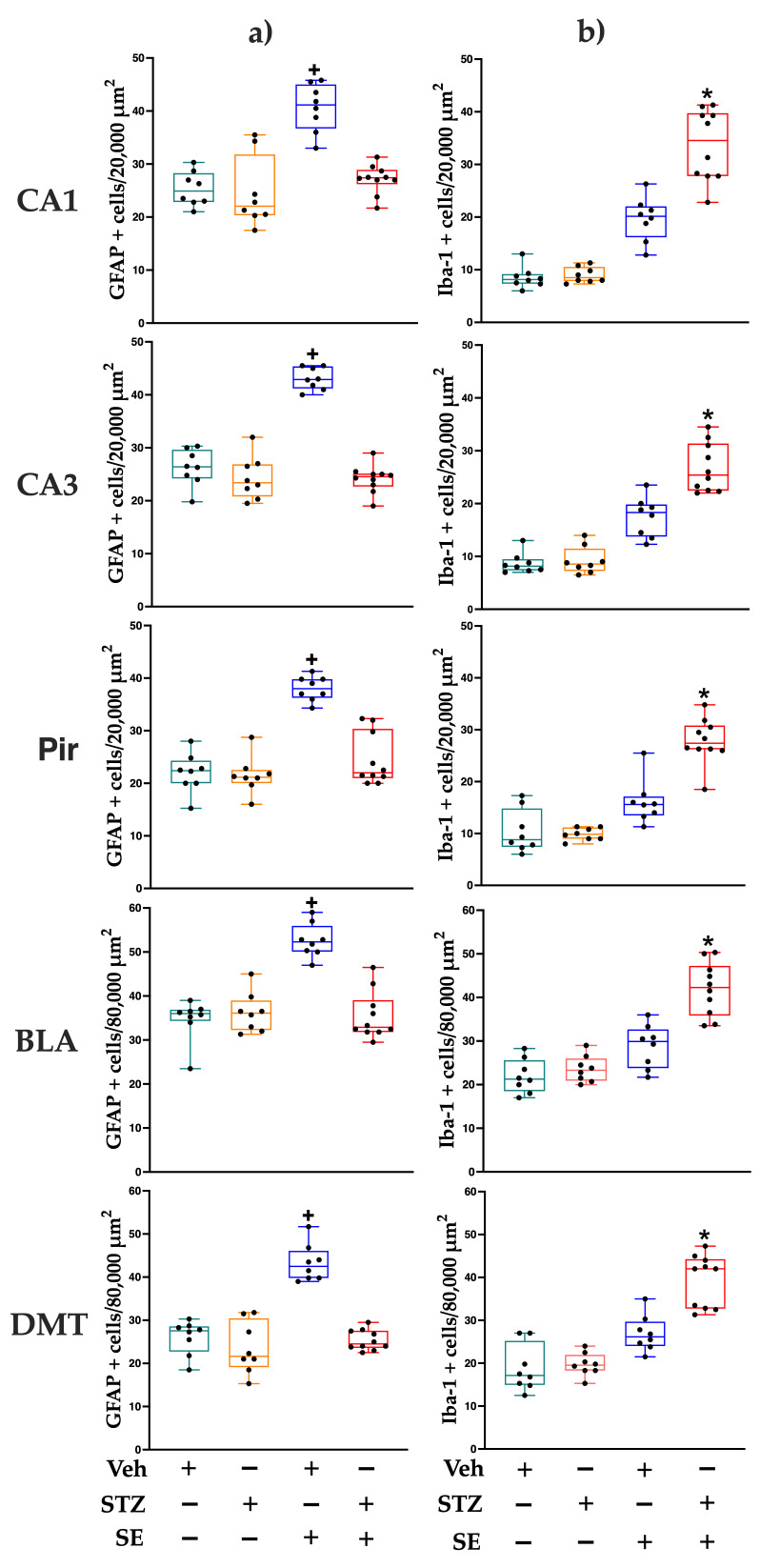
Number of GFAP-(**a**) or Iba-1-immunoreactive cells (**b**) in CA1 and CA3 regions of the dorsal hippocampus, piriform cortex (Pir), basolateral amygdala (BLA), and dorsomedial thalamus (DMT) of rats treated with streptozotocin (STZ) or vehicle (Veh), with or without *status epilepticus* (SE). Data represent medians and interquartile ranges; whiskers indicate minimum and maximum values for each group. Individual data points represent each rat (n = 8 Veh, 8 STZ, 8 Veh + SE and 10 STZ + SE). + *p* < 0.05 vs. Veh, STZ and STZ + SE; * *p* < 0.05 vs. Veh, STZ, and Veh + SE.

**Figure 4:**
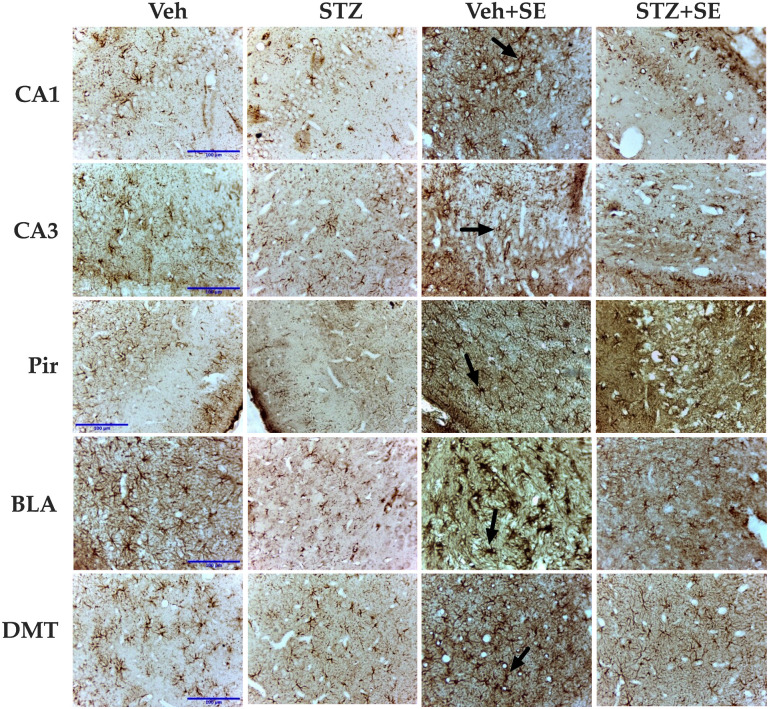
Representative photomicrographs (40×) of GFAP-immunoreactive cells in CA1 and CA3 regions of the hippocampus, piriform cortex (Pir), basolateral amygdala (BLA), and dorsomedial thalamus (DMT) from rats treated with vehicle (Veh) or streptozotocin (STZ), with or without *status epilepticus* (SE). The Veh + SE group exhibits a greater number of astrocytes in all evaluated regions compared with all other groups. Arrows indicate GFAP-immunoreactive cells. Scale bar = 100 µm.

When we evaluated whether the glial response in the STZ + SE group differed from that expected from the independent effects of STZ treatment and SE, the two-way ANOVA revealed significant effects of STZ treatment on the number of GFAP-immunoreactive cells in all analyzed regions (CA1: F(1,30) = 20.83, *p* < 0.001, ηp^2^ = 0.418; CA3: F(1,30) = 81.16, *p* < 0.001, ηp^2^ = 0.759; Pir: F(1,30) = 30.74, *p* < 0.0001, ηp^2^ = 0.481; BLA: F(1,30) = 23.29, *p* < 0.0001, ηp^2^ = 0.432; DMT: F(1,30) = 16.60, *p* < 0.0001, ηp^2^ = 0.615). SE also significantly affected GFAP-immunoreactive cell counts (CA1: F(1,30) = 28.47, *p* < 0.0001, ηp^2^ = 0.530; CA3: F(1,30) = 37.98, *p* < 0.001, ηp^2^ = 0.666; Pir: F(1,30) = 47.66, *p* < 0.0001, ηp^2^ = 0.632; BLA: F(1,30) = 21.97, *p* < 0.0001, ηp^2^ = 0.481; DMT: F(1,30) = 47.98, *p* < 0.0001, ηp^2^ = 0.584). A significant interaction between STZ treatment and SE was found in all regions (CA1: F(1,30) = 19.97, *p* < 0.0001, ηp^2^ = 0.364; CA3: F(1,30) = 58.15, *p* < 0.001, ηp^2^ = 0.674; Pir: F(1,30) = 33.03, *p* < 0.0001, ηp^2^ = 0.451; BLA: F(1,30) = 35.53, *p* < 0.0001, ηp^2^ = 0.524; DMT: F(1,30) = 11.17, *p* < 0.05, ηp^2^ = 0.480). Post hoc analysis revealed an increased number of GFAP-immunoreactive cells only in the Veh + SE group compared with the Veh, STZ, and STZ + SE groups, as shown in Figure 3 and Figure 4. These results indicate that, although both STZ and SE independently modulate astrocytic activity, their coexistence does not produce a synergistic effect on glial activation.

**Figure 5:**
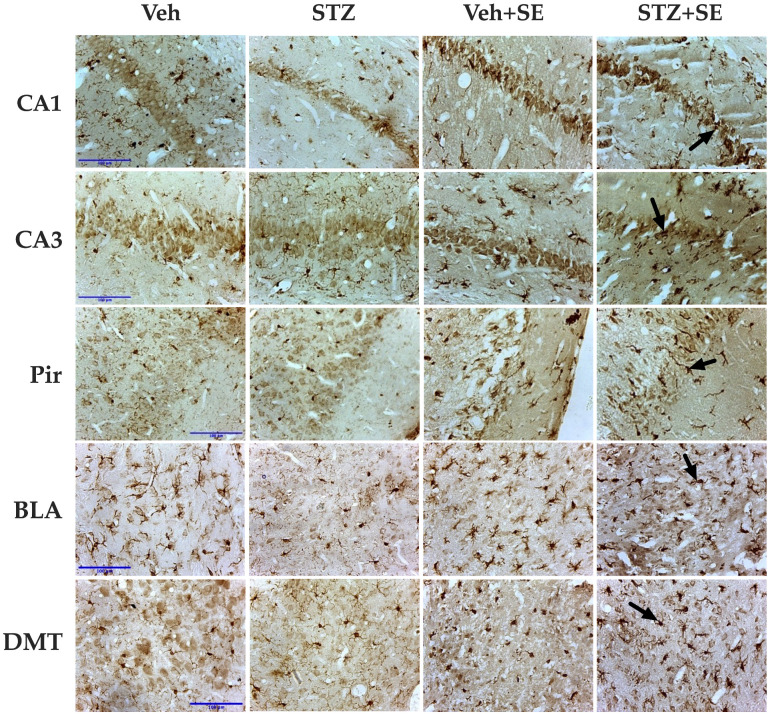
Representative photomicrographs (40×) of Iba-1-immunoreactive cells in CA1 and CA3 regions of the hippocampus, piriform cortex (Pir), basolateral amygdala (BLA), and dorsomedial thalamus (DMT) from rats treated with vehicle (Veh) or streptozotocin (STZ), with or without *status epilepticus* (SE). The STZ + SE group exhibits a higher number of microglial cells compared with all other groups. Arrows indicate to Iba-1-immunoreactive cells. Scale bar = 100 µm.

Regarding the microglial, significant main effects of STZ treatment (CA1: F(1,30) = 32.29, *p* < 0.0001, ηp^2^ = 0.433; CA3: F(1,30) = 24.07, *p* < 0.0001, ηp^2^ = 0.358; Pir: F(1,30) = 25.12, *p* < 0.05, ηp^2^ = 0.382; BLA: F(1,30) = 26.52, *p* < 0.0001, ηp^2^ = 0.404; DMT: F(1,30) = 21.70, *p* < 0.05, ηp^2^ = 0.342) and SE (CA1: F(1,30) = 151.94, *p* < 0.0001, ηp^2^ = 0.823; CA3: F(1,30) = 128.40, *p* < 0.0001, ηp^2^ = 0.800; Pir: F(1,30) = 70.61, *p* < 0.0001, ηp^2^ = 0.733; BLA: F(1,30) = 66.21, *p* < 0.0001, ηp^2^ = 0.662; DMT: F(1,30) = 75.95, *p* < 0.0001, ηp^2^ = 0.693) were observed across all analyzed regions, with significant interaction between STZ and SE in CA1 (F(1,30) = 12.79, *p* < 0.05, ηp^2^ = 0.400), CA3 (F(1,30) = 7.54, *p* < 0.0001, ηp^2^ = 0.307), Pir (F(1,30) = 10.67, *p* < 0.05, ηp^2^ = 0.425), BLA (F(1,30) = 7.94, *p* < 0.05, ηp^2^ = 0.290), and DMT (F(1,30) = 7.71, *p* < 0.05, ηp^2^ = 0.280). This interaction indicates that the coexistence of T2DM and SE potentiated microglial activation beyond the effect of each condition alone. Post hoc analyses confirmed a marked increase in the number of Iba-1–immunoreactive cells in all regions of the STZ + SE group compared with the Veh, STZ, and Veh + SE groups (CA1, CA3, Pir, and BLA, *p* < 0.0001; DMT, *p* < 0.001), as shown in Figure 3 and Figure 5.

## 4. Discussion

Inadequate glycemic control in T2DM may increase seizure susceptibility and aggravate brain damage. Our study found that rats with T2DM developed a more severe form of SE characterized by widespread neurodegeneration, cell loss, microgliosis and attenuated glia response.

STZ-injected rats that developed T2DM exhibited longer latencies to both generalized seizures and SE onset, consistent with previous observations [22]. However, our findings differ from those of Huang et al. [20], who induced SE in adult rats with T1DM and observed shorter seizure latencies compared with controls. This discrepancy likely stems from distinct pathophysiological mechanisms underlying each DM model, which may imply different mechanisms influencing seizure susceptibility. We also found that STZ-induced T2DM rats experienced a higher number of stage V seizures, similar to previous results from kainate or lithium–pilocarpine models in mice and rats with T1DM [20,21]. Several mechanisms linked to chronic hyperglycemia in T2DM, including oxidative stress, blood–brain barrier disruption, altered calcium homeostasis, and decreased activity of ATP-sensitive potassium channels, likely converge to amplify seizure severity [33,34,35,36,37].

Our experimental data align with clinical observations by Huang et al. [11], who conducted a prospective study on adults with newly diagnosed unprovoked seizures, both with and without DM. The authors followed participants for two years to assess seizure frequency and severity and found higher rates of seizure clustering and SE in diabetic individuals. Additionally, they observed that elevated glycosylated hemoglobin levels, which reflect poor long-term glycemic control, were positive associated with seizure risk.

On the other hand, the results of our study showed that T2DM exacerbated SE-induced neurodegeneration, neuronal loss and tissue damage across multiple brain regions, including the hippocampus, piriform cortex, thalamus and amygdala. These findings agree with classic descriptions by Turski et al. [28], who reported extensive neuronal degeneration, edema, neuropil disruption and swelling within 24 h of pilocarpine-induced SE. Likewise, other studies, although primarily focused on the identification of necrotic neuronal cell death following pilocarpine- or kainic acid-induced SE, also present H&E-stained histological images of the piriform cortex, hippocampus, and other brain regions in which tissue loss comparable to our findings can be discerned [29,32]. These areas, characterized by the absence of cellular or neuropil staining, appear alongside acidophilic cells 24 h after SE. Furthermore, these authors describe extensive edema and neuropil disruption as neuropathological alterations induced by SE in the rat brain. The pronounced neuronal and tissue loss in our STZ-SE group suggest that persistent hyperglycemia compromises brain integrity, rendering tissue more vulnerable to excitotoxic injury. While the precise mechanisms remain uncertain, oxidative stress, altered blood–brain barrier and calcium homeostasis, neuroinflammation or metabolism alterations characteristic of T2DM [38,39,40] likely work in concert to increase both neuronal excitability and tissue susceptibility

Regarding the glia response, diabetic rats subjected to SE did not show the expected increase in astrocyte number in the examined regions. This stands in sharp contrast to the robust gliosis seen in Veh + SE rats. This attenuated astrocytic reactivity mirrors findings by Kalani and collaborators [31], who observed reduced GFAP immunoreactivity in mice with T1DM following ischemic injury. Similar reductions in GFAP expression have been reported in STZ-induced diabetic rats despite stable astrocyte counts [41]. Additionally, exposure to high glucose in vitro has been shown to inhibit astrocytic proliferation and migration [42]. Given the critical role of astrocytes in glutamate clearance and neuroprotection [43,44], reduced astrocytic activity under hyperglycemic conditions may further aggravate the neuronal degeneration observed in T2DM. Metabolic disturbances may reshape the neural microenvironment in ways that favor astrocytic dysfunction and impair glial-neuronal homeostasis, not only exacerbating neuronal damage but also promoting astrocytic death, as observed in rats with T1DM [45].

In contrast, we found marked increase in the number of microglial cells in diabetic rats after SE across all analyzed brain regions. In the context of brain disease or injury, such as SE, inflammatory factors interferon gamma or tumor necrosis factor alpha activate microglia and increase their proliferation in response to damage [46]. Excessive polarization toward the M1 (pro-inflammatory) phenotype, relative to the M2 (neuroprotective) state, may amplify neuronal injury [47]. The enhanced microgliosis observed here likely reflects a maladaptive neuroinflammatory response secondary to chronic hyperglycemia and may contribute to neuronal loss and neurodegeneration detected in diabetic rats.

This study has two main limitations. First, we evaluated only male rats, so potential sex-dependent effects remain unexplored. Second, the interpretation of tissue loss in epilepsy models remains debated and should be approached with caution. Nevertheless, our results align with previously documented neuropathological alterations following SE [28,29,31,32] and reinforce the notion that T2DM worsens these outcomes. Future work combining ultrastructural, metabolic, and electrophysiological analyses under normoglycemic and hyperglycemic conditions should help clarify how systemic metabolic disturbances translate into local neurophysiological vulnerability.

## 5. Conclusions

Our data suggest that T2DM in male adult rats exacerbates seizure severity and increases neuronal injury in limbic regions, including the hippocampus, amygdala, thalamus, and piriform cortex. Interestingly, T2DM markedly enhanced microglial activation while reducing glia reactivity, suggesting an altered glial balance in the diabetic brain. These findings provide preclinical evidence for the relationship between diabetes and epilepsy. Future studies should address how chronic hyperglycemia disrupts the brain’s excitatory homeostasis, which may help explain the increased seizure susceptibility observed in diabetic patients.

## Data Availability

The raw data supporting the conclusions of this article will be made available by the authors on request.

## Abbreviations

The following abbreviations are used in this manuscript:

SE	*Status epilepticus*
T2DM	Type 2 diabetes mellitus
STZ	Streptozocin
P	Postnatal day
i.p.	Intraperitoneal
s.c.	Subcutaneous
DM	Diabetes mellitus
T1DM	Type 1 diabetes mellitus
Veh	Vehicle
PB	Phosphate buffer
F-JB	Fluoro-Jade B
H&E	Hematoxylin and eosin staining
PBT	Phosphate-buffer containing 1% triton
GFAP	Glial fibrillary acidic protein
Iba-1	Ionized calcium-binding adapter molecule 1
CA1	CA1 hippocampal field
CA3	CA3 hippocampal field
Pir	Piriform cortex
BLA	Basolateral amygdala nucleus
DMT	Dorsomedial thalamic nucleus
ROI	Region of interest
ANOVA	Analysis of variance
